# Application of nanoindentation technology in testing the mechanical properties of skull materials

**DOI:** 10.1038/s41598-022-11216-6

**Published:** 2022-05-24

**Authors:** Jia-Wen Wang, Kai Yu, Man Li, Jun Wu, Jie Wang, Chang-Wu Wan, Chao-Lun Xiao, Bing Xia, Jiang Huang

**Affiliations:** 1 School of Forensic Medicine, Guizhou Medical University, Guiyang, 550004 China; 2 Department of Forensic Pathology, College of Forensic Medicine, Xi’an Jiaotong University, Xi’an, 710061 China; 3 Basic Medical College, Guizhou Medical University, Guiyang, 550004 China

**Keywords:** Structural biology, Anatomy, Medical research, Mathematics and computing, Physics

## Abstract

Three-point bending test, compression test and tensile test can detect the mechanical properties of the whole layer of skull, but cannot detect the mechanical properties of the inner plate, the diploe and the outer plate of the skull. In this study, nanoindentation technology was applied to detect mechanical properties of micro-materials of the skull, and differences in micro-mechanical properties of the inner, diploe and outer plates of the skull and cranial suture of human carcasses at different ages were analyzed. The differences in hardness (HIT) and modulus of elasticity (E) were statistically significant among different age groups (*P* < 0.01). In terms of structure, the E of diploe was higher than that of other structures, while HIT had no significant statistical difference. In terms of location, both HIT and E showed that left frontal (LF) was significantly higher than coronal suture (CS). The above results were consistent with the multi-factor ANOVAs. In addition, the multi-factor ANOVAs further explained the interaction of HIT and E with age, location and structure. It was believed that the nanoindentation technique could be used to analyze laws of micromechanical properties of different structures of human cadaveric skull and cranial suture.

## Introduction

Traumatic brain injury (TBI) is common in forensic actual cases which accounts for the largest proportion of the causes of traumatic death, and it is often accompanied by skull fracture^1,2^. Studies have shown that finite element models have been developed and applied in various human injury mechanism analysis^3,4^. The material parameters of finite element modeling of skull usually derive from cadaveric skull experiments. The three-point bending experiment using skull fragments has shown differences in biomechanical parameters of different parts of the skull and the cranial suture in corpses at different ages, thus indicating that the values of the skull and the cranial suture in different parts should be respectively assigned when the finite element model of the skull was constructed^5,6^. In addition, the skull bones such as frontal, temporal, parietal, and occipital bones are similar to the “sandwich” structure, consisting of the outer plate, diploe, and inner plate^7^. The outer and inner plates are osteon-dense, and the diploe is cancellous, which microstructures vary greatly. The osteon-dense bone consists of a compact and regularly arranged osteon, while the cancellous bone consists of trabecular bone, which is loose and porous. At present, most finite element models regard the dense/cancellous bone of skull in all parts as a single structure composed of uniform materials. However, studies have shown that the geometry and spatial arrangement of the osteon and trabecular meshwork have great effects on the mechanical properties of the bone^8–10^, which indicates that the biomechanical properties of different parts/layers of the skull may vary. Previous studies have shown differences in mechanical parameters of materials between the outer and inner plates of animal skulls^11^. Nonetheless, the above studies have not detected the mechanical parameters for skull diploe. Burket et al. found age-related changes in elastic modulus and hardness in animals, manifesting as a sharp increase of the two during rapid bone growth, tending to stabilize during sexual maturation^12^. However, whether there are differences in biomechanical properties of inner plate, outer plate, and diploe between the skulls and cranial sutures in humans at different ages have not yet been reported. If the above biomechanical parameters were known, the relationship between the shape of skull fracture and injury risk factors such as the magnitude and direction of force could be analyzed more deeply.

The conventional detection methods for biomechanical parameters of the skull, such as a complete skull impact test and a three-point bending test, can hardly accurately detect the skulls with different layers (inner plate, outer plate, and diploe) and can even damage most of the whole sample. Studies have shown that nanoindentation technique has strong advantages in measuring the mechanical properties of materials within different micro-regions, as it can meet the requirements of in-situ and non-destructive testing and can also be used to test samples with small sizes or different shapes^13–16^. The material mechanical parameters of human trabecular meshwork such as humerus and femur have been measured using nano-indentation technology. Other studies have shown that nanoindentation technology has a unique value in measuring the bone parameters in tiny areas such as trabecular bone^17,18^. Therefore, we believed that this method could be used to detect the mechanical parameters of materials in various micro-regions of the skull.

In this study, we explored the feasibility of using nanoindentation to detect the biomechanical parameters in human skulls at different ages, including different parts (frontal bone and coronal suture), and different layers (inner plate, outer plate, and diploe). The purpose is to provide the corresponding reference basis for the construction of a more precise and comprehensive human skull finite element model, and make the model more accurate in the mechanism analysis of craniocerebral injury. At the same time, our study is also important for a comprehensive understanding of the biomechanical research on skull fracture at the micro-level.

## Materials and methods

### Sample collection

Skull samples (n = 5) were collected from autopsy cases conducted at the School of Forensic Medicine/Forensic Medicine Identification Center of Guizhou Medical University during 2019 (Table 1). All cranial donors were male, and they were divided into five groups according to age, i.e., infant, young child, school-age child, middle-aged and elderly group, with one case per group. Complete personal information such as gender, age, height, and a clear cause of death were available for each skull donor. Note chosen skull donors did not have a skull related complication. Since the freezing process of human hard tissues has little effect on their mechanical properties, the obtained skull samples were unified frozen and stored at the − 20 °C refrigerator for future examination^19,20^.

**Table 1:** Skull sample provider information.

Group	Gender	Age	Height (cm)	Death
Baby	Male	17 days	51	Pulmonary infection with hyperbilirubinemia
child	Male	3 years	87	Bronchial lumen obstruction
School-age children	Male	8 years	123	Viral myocarditis
Middle-aged	Male	51 years	178	Sudden cardiac death
Old age	Male	77 years	167	Coronary heart disease

### Sample preparation

**Figure 1:**
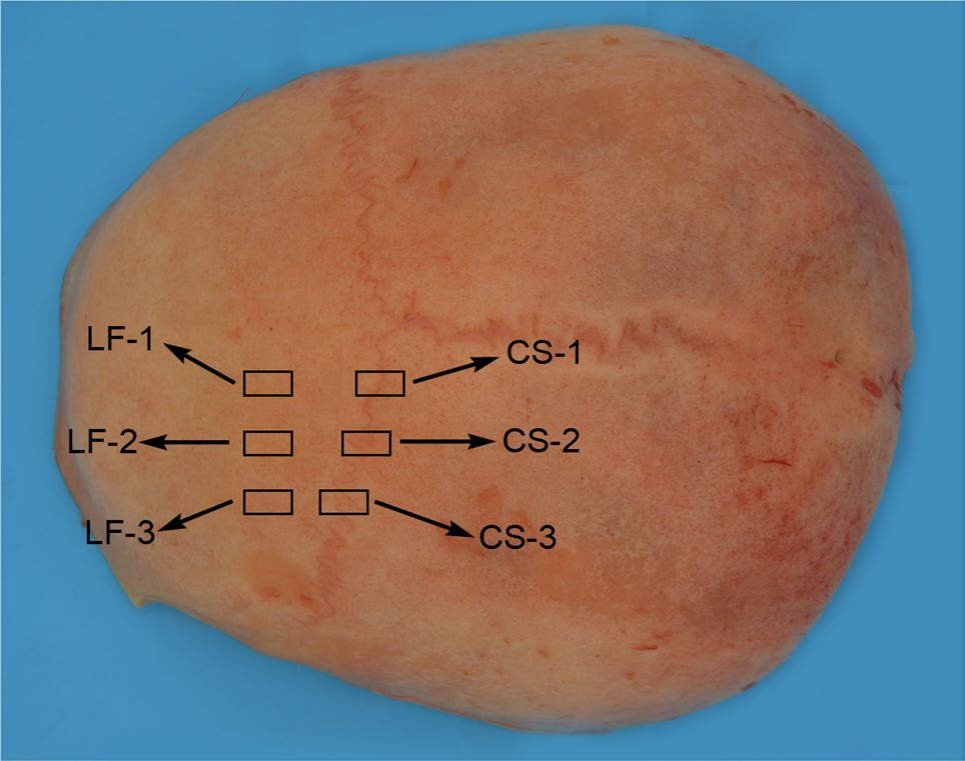
Material drawing of each part of the skull.

**Figure 2:**
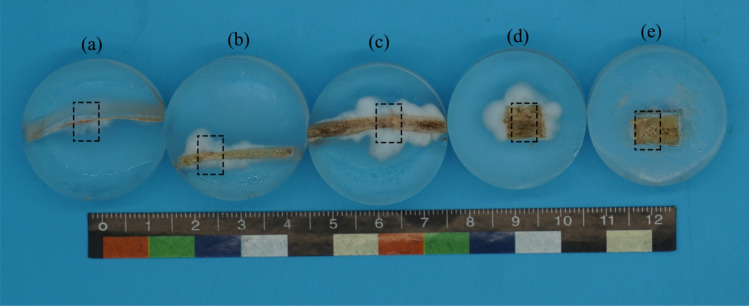
Physical diagram of CS samples at different ages. Samples (**a**, **b**, **c**, **d**, and **e**) were 0.047, 3, 8, 51, and 77 years of age, respectively.

As shown in Fig. 1, for each skull sample (n = 5), three left frontal (LF) and three coronal suture (CS) pieces with a size of approximately (1~3) cm × 1 cm were cut using an electric cutter. LF-1, LF-2, and LF-3 labeled on LF represented the outer plate (O), inner plate (I), and diploe (D) experimental specimens for nanoindentation detection, respectively. Similarly, each structure of CS was labeled as CS-1, CS-2, and CS-3. Epoxy resin and toughening curing agent were uniformly mixed with a mass ratio of 100:40 and then poured into a cylindrical soft silica gel mold with an inner diameter of 30 mm. Thawed samples were placed in the prepared gel with the experimental side down at room temperature until completely solidified. Next, low-mesh to high-mesh metallographic sandpapers (mesh numbers: 800, 1200, 1500, 2000, and 4000) were sequentially used to grind and polished on a metallographic sample polishing machine (MPD-1) at a rotating speed of 500 r/min until there were no obvious scratches on the surface of the samples. Until all areas on the experimental surface of the sample could be clearly observed under the microscope with the same magnification (Fig. 2).

### Thickness measurement

Prior to the nanoindentation measurements, thickness of the considered sections was measured under the microscope configured to the nanoindentor. The thickness of the inner plate, the diploe and the outer plate of the sections were obtained as well as the total thickness. The total thickness, the thickness of the inner plate, the diploe and the outer plate were obtained into three values. The mean value and the standard deviation were obtained through calculation.

### Nanoindentation experiment

**Figure 3:**
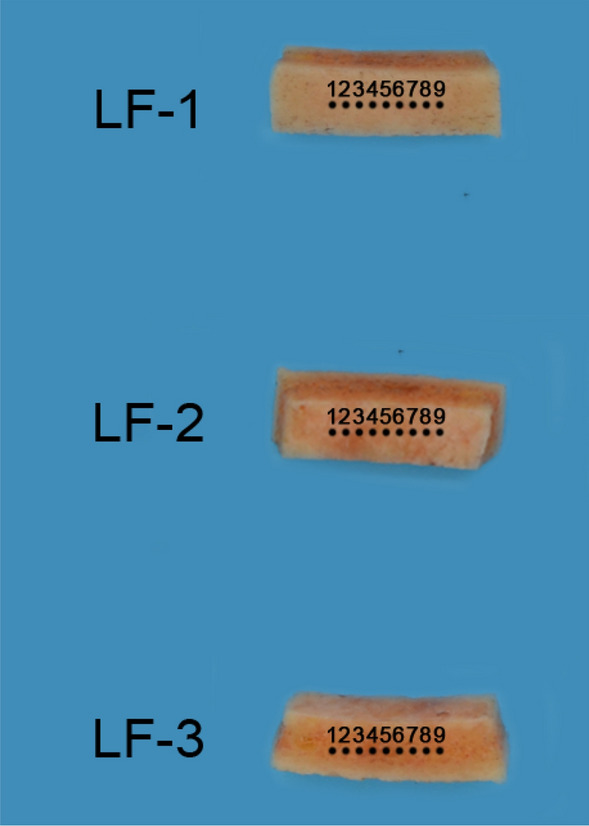
Bitmap of nanoindentation points of inner plate, diploe and outer plate of the LF.

Experiments were performed using an NHT3 (Anton-Paar, PESEUX, Switzerland) nanoindentation apparatus. The tip of the ram was loaded into the sample at 40 mN/min for 10 s at a maximum load of 20 mN and then loaded out at 40 mN/min. As shown in Fig. 3, nine points were selected on the the outer plate and the inner plate surface of the skull (LF-1, LF-2), and the midpoint between the outer plate and the inner plate surface of the skull (LF-3, where is cancellous bone) for nano indentation detection. Of the above nine points, the distance between adjacent points is 100 μm. In the actual detection process, considering the weak compression ability of CS and the limitation of nanoindentation technology, some selected detection indentations were located in CS, while most of them were on both sides of CS. During the test, the computer detection software automatically outputs loading and contact areas to calculate Brinell hardness (HIT in MPa) and modulus of elasticity (E in GPa). The method proposed by Oliver and Pharr et al. was used to obtain the modulus of elasticity (EIT in GPa)^21^ (assuming v = 0.3 poisson's ratio).

### Statistical analysis

Descriptive results were presented primarily as means and standard deviation (SD). All statistical calculations were performed by IBM SPSS statistical software (SPSS, Version 25, IBM, Armonk, New York). *P* < 0.05 was considered statistically significant. One-way and multi-way ANOVAs were used to test the main effect contributions of age, structure, and location, as well as the interaction between E and HIT.

### Ethical considerations

This study was approved by the Ethics Committee of Guizhou Medical University (approval 2021 No.1). Informed consent was obtained from the parents and/or legal guardians for the use of the skull specimen in this study. All experimental procedures and methods were performed in accordance with approved guidelines and standards applicable to the forensic context.

## Results

### Total thickness of each layers of LF and CS

**Figure 4:**
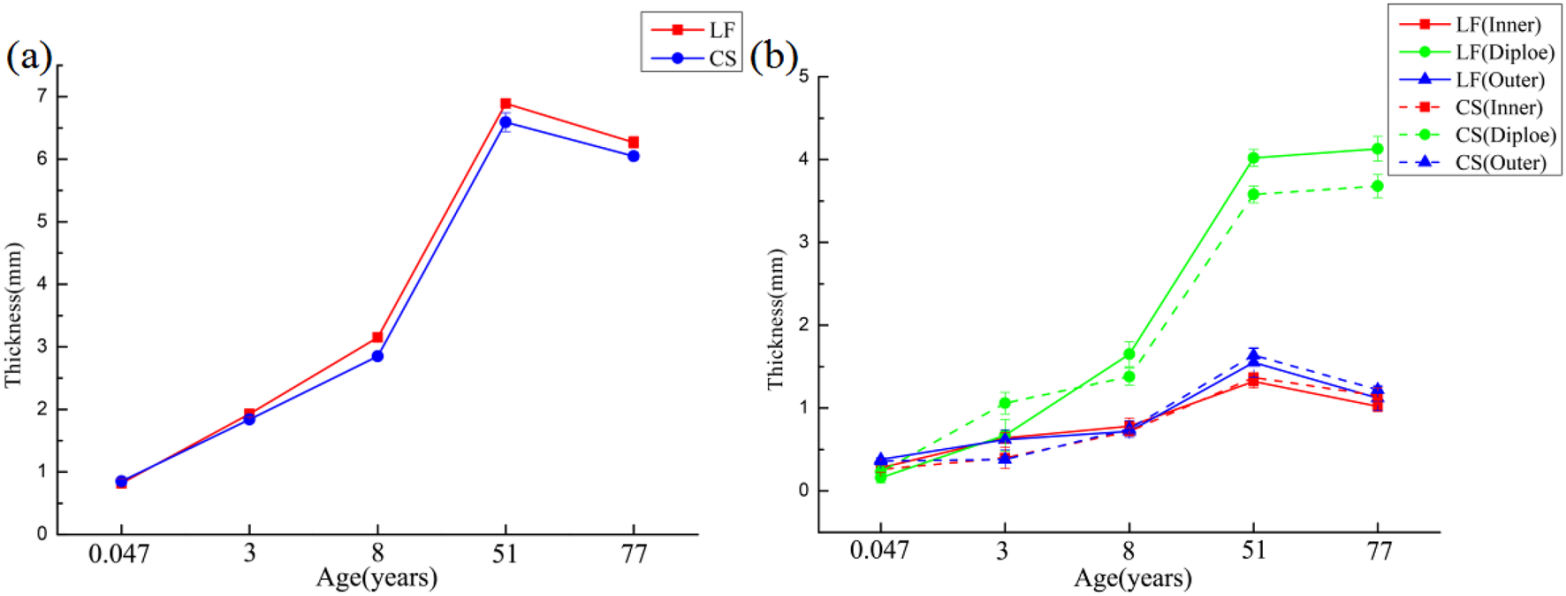
Change trend diagram of thickness of total and different layers of LF and CS at different ages. The (**a**) is the thickness of the whole skull, and (**b**) is the thickness of each layer of the skull inner plate, the diploe and the outer plate.

With age increasing, the total thickness of the LF and CS gradually increased, reaching the maximum in the 51 years of age group and then decreasing slightly (Fig. 4a). The thickness of LF was higher than that of CS (except for 0.047 years of age group).

The results of different layers of LF and CS showed that the thicknesses of the inner plate, diploe and outer plate tended to increase with age, and the thicknesses of LF and CS diploe were higher than those of the inner plate and outer plate (Fig. 4b).

### Effects of age, layers, and location on HIT and E

**Figure 5:**
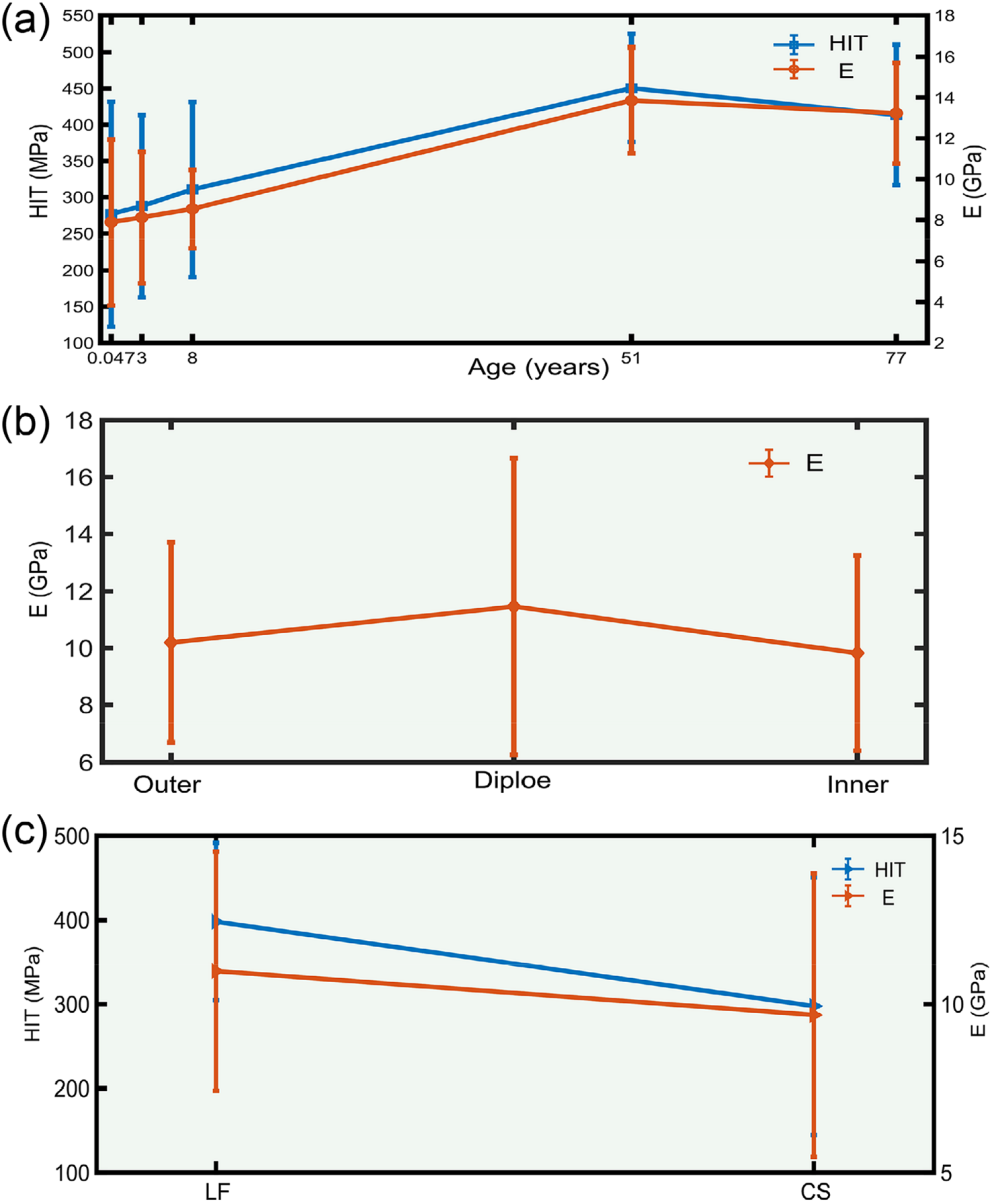
One-way analysis of variance of HIT and E based on different ages, layers and locations. The (**a**) is the HIT and E changing trend of LF and CS at different ages; (**b**) is the E difference of different structures of inner plate, diploe and outer plate; (**c**) is the difference comparison of different parts (LF and CS).

Descriptive results are mainly reported as the mean and the standard deviation (SD) (Tables 2, 3). Overall, results showed that there are statistical differences in HIT (*P* < 0.001) and E (*P* < 0.001) among the considered age groups. In Fig. 5a, the left Y-axis represents HIT, and the right Y-axis represents E. HIT and E showed an increasing trend before 51 years of age and a slightly decreasing trend after 51 years of age. HIT between the inner, outer plates and diploe were not correlated (*P* = 0.835), while E exhibited a significant trend (*P* = 0.056) and diploe E were higher than that of the outer and inner plates (Fig. 5b). There were significant differences in HIT and E of each site between groups (*P* < 0.05), and HIT and E of LF were significantly higher compared to those of CS (Fig. 5c).

**Table 2:** HIT single-factor ANOVA.

Group	Class	n	Mean	SD	*P* value
Ages(years)	0.047	46	276.918	154.726	< 0.001
	3	46	287.871	125.188	
	8	46	310.827	120.336	
	51	46	450.403	74.097	
	77	46	413.329	96.639	
Layers	O	90	354.629	123.636	= 0.835
	D	50	343.510	131.268	
	I	90	343.532	151.063	
Locations	LF	115	398.133	93.506	< 0.001
	CS	115	297.606	152.887	

SD: standard deviation; n: number; O: outer plate; D: diploe; I: inner plate; LF: left frontal; CS: coronal suture (Tables 3, 6 and 7 are the same).

**Table 3:** E single-factor ANOVA.

Group	Class	n	Mean	SD	*P* value
Ages (years)	0.047	46	7.888	4.055	< 0.001
	3	46	8.125	3.209	
	8	46	8.540	1.904	
	51	46	13.855	2.591	
	77	46	13.232	2.467	
Layers	O	90	10.199	3.508	= 0.056
	D	50	11.465	5.200	
	I	90	9.825	3.416	
Locations	LF	115	10.982	3.547	= 0.011
	CS	115	9.674	4.203

### Effect of combination of age, layers and location on HIT and E

**Figure 6:**
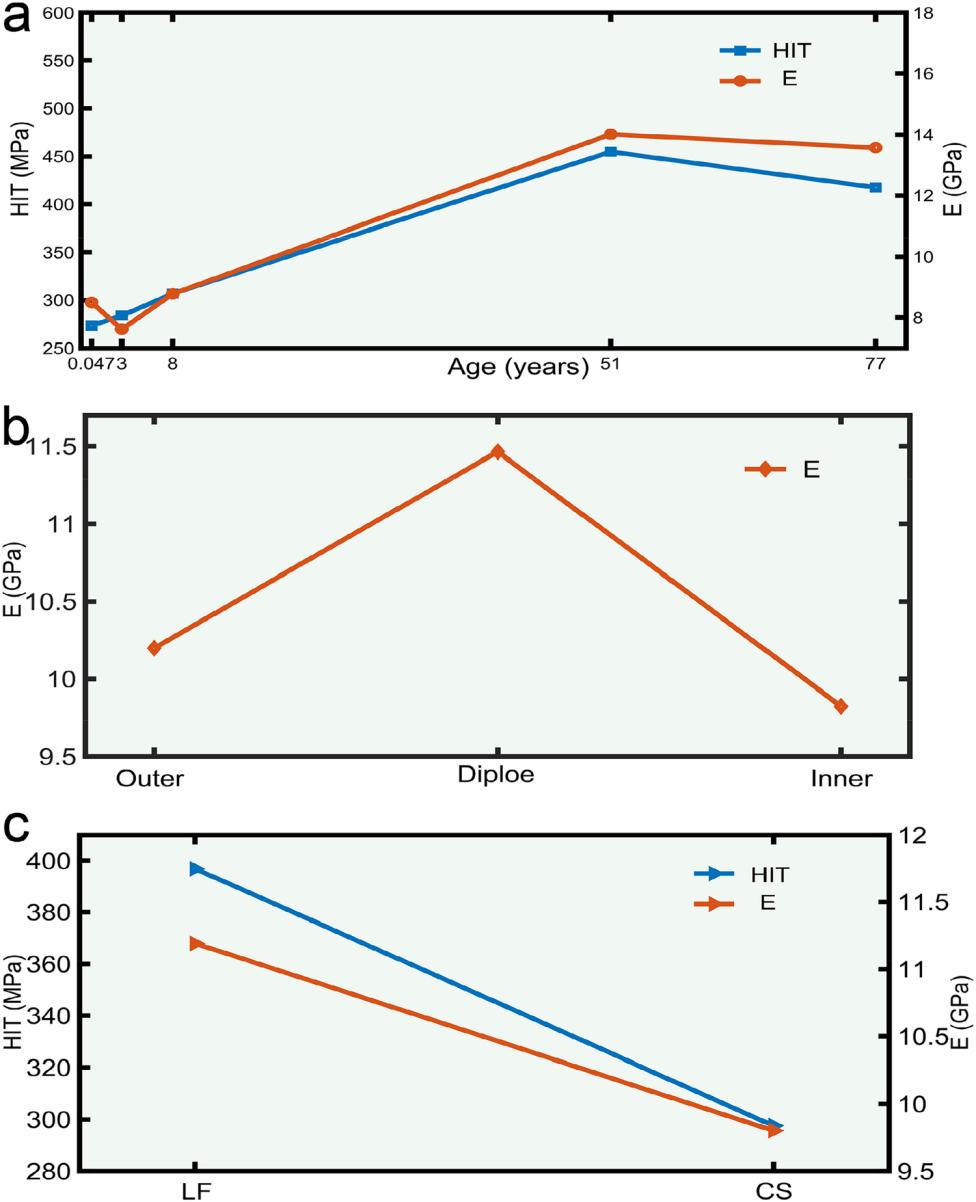
Multi-way analysis of variance of HIT and E based on different ages, layers and locations. The (**a**) is the HIT and E changing trend of LF and CS at different ages; (**b**) is the E difference of different layers of inner plate, diploe and outer plate; (**c**) is the difference comparison of different parts (LF and CS).

**Figure 7:**
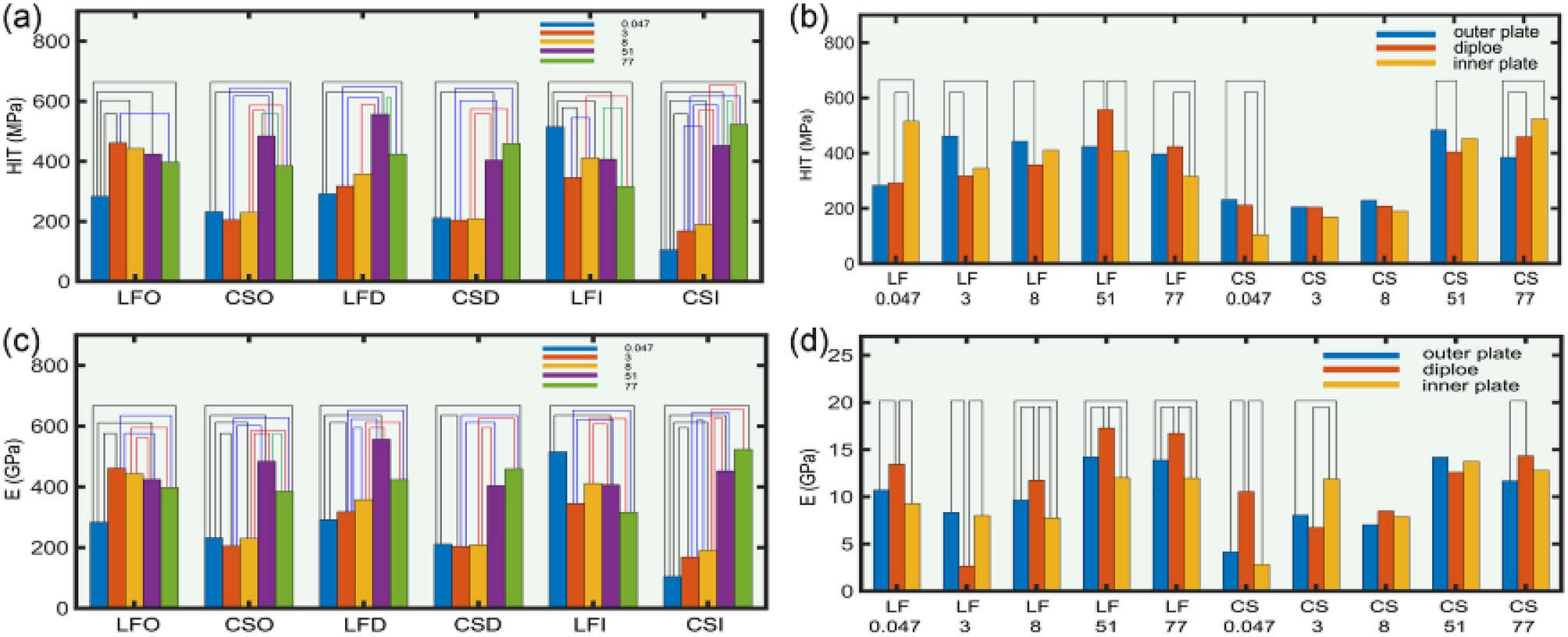
Multi-way analysis of variance of HIT and E based on different ages, layers and locations. The (**a**) and (**c**) show the statistical description of HIT and E of ages at the same layers and location. The (**b**) and (d) show the statistical description of HIT and E of location at the same layers and ages. And the group with statistical significance are connected by straight lines.

Multi-factor ANOVAs determined the main effect contribution of age, layers and location and their interactions on E and HIT. In Table 4, HIT of age (*P* < 0.001) and location (*P* < 0.001) had statistically significant, while HIT of the layers had no correlation (*P* = 0.487). In Table 5, E of age (*P* < 0.001), layers (*P* < 0.001) and location (*P* < 0.001) had statistically significant. The same age and structure, HIT and E statistical analysis of location were shown in Tables 6 and 7, respectively. Same as the result of single-factor ANOVAs, HIT showed an upward trend before 51 years of age and a downward trend after 51 years of age, and difference from the results of single-factor ANOVAs, E decreased from 0.047 to 3 years of age, then increased to 51 years of age, and finally continued to decrease to 77 years of age (Fig. 6a). Diploe E was higher than that of the outer and inner plates (Fig. 6b). And HIT and E of LF were significantly higher than that of CS (Fig. 6c). Figure 7 showed the same layers and location, age and location, statistical description of the age, layers were displayed. HIT and E for both CSD and LFD showed results consistent with a one-way analysis of variance that were maximal in the 51 years of age group (Fig. 7a,c). The same age and location, we found higher E of plate in the 0.047, 8, 51, and 77 years of age groups of LF, and in the 0.047, 8, and 77 years of age groups of CS than in the inner and outer plates, which was consistent with the results of a one-way analysis of variance (Fig. 7d).

**Table 4:** HIT tests of between-subjects effects.

Group	*P* value
Ages (years)	< 0.001
Layers	= 0.487
Locations	< 0.001
Ages (years) and layers	< 0.001
Ages (years) and locations	< 0.001
Layers and locations	= 0.642
Ages (years), layers and locations	< 0.001

R^2^ = 0.78.

**Table 5:** E tests of between-subjects effects.

Group	*P* value
Ages (years)	< 0.001
Layers	< 0.001
Locations	< 0.001
Ages (years) and layers	< 0.001
Ages (years) and locations	< 0.001
Layers and locations	< 0.001
Ages (years), layers and locations	< 0.001

R^2^ = 0.798.

**Table 6:** HIT comparison among different locations.

Age (years)	Layers	LA	Mean	LB	MD (LA-LB)	*P* value
0.0047	O	LF	283.491	CS	51.651	= 0.111
		CS	231.840	LF	− 51.651	= 0.111
	D	LF	291.634	CS	80.002	= 0.066
		CS	211.632	LF	− 80.002	= 0.066
	I	LF	515.377	CS	410.318*	< 0.001
		CS	105.059	LF	− 410.318*	< 0.001
3	O	LF	461.672	CS	256.132*	< 0.001
		CS	205.540	LF	− 256.132*	< 0.001
	D	LF	317.662	CS	113.442*	= 0.009
		CS	204.220	LF	− 113.442*	= 0.009
	I	LF	345.619	CS	177.042*	< 0.001
		CS	168.577	LF	− 177.042*	< 0.001
8	O	LF	443.214	CS	213.597*	< 0.001
		CS	229.618	LF	− 213.597*	< 0.001
	D	LF	357.784	CS	149.212*	= 0.001
		CS	208.572	LF	− 149.212*	= 0.001
	I	LF	410.998	CS	220.799*	< 0.001
		CS	190.199	LF	− 220.799*	< 0.001
51	O	LF	424.166	CS	− 60.316	= 0.063
		CS	484.481	LF	60.316	= 0.063
	D	LF	556.712	CS	152.764*	= 0.001
		CS	403.948	LF	− 152.764*	= 0.001
	I	LF	407.073	CS	− 45.567	= 0.159
		CS	452.640	LF	45.567	= 0.159
77	O	LF	397.442	CS	12.614	= 0.696
		CS	384.828	LF	− 12.614	= 0.696
	D	LF	423.760	CS	− 35.420	= 0.414
		CS	459.180	LF	35.420	= 0.414
	I	LF	316.234	CS	− 207.309*	< 0.001
		CS	523.543	LF	207.309*	< 0.001

MD: mean different; LA: location A; LB: location B; *: significant differences (Table 7 are the same).

**Table 7:** E comparison among different locations.

Age (years)	Layers	LA	Mean	LB	MD (LA-LB)	*P* value
0.0047	O	LF	10.720	CS	6.570*	< 0.001
		CS	4.150	LF	− 6.570*	< 0.001
	D	LF	13.463	CS	2.928*	= 0.015
		CS	10.534	LF	− 2.928*	= 0.015
	I	LF	9.294	CS	6.472*	< 0.001
		CS	2.822	LF	− 6.472*	< 0.001
3	O	LF	8.336	CS	0.269	= 0.764
		CS	8.067	LF	− 0.269	= 0.764
	D	LF	2.671	CS	− 4.068*	= 0.001
		CS	6.739	LF	4.068*	= 0.001
	I	LF	8.009	CS	− 3.880*	< 0.001
		CS	11.889	LF	3.880*	< 0.001
8	O	LF	9.654	CS	2.600*	= 0.004
		CS	7.054	LF	− 2.600*	= 0.004
	D	LF	11.774	CS	3.278*	= 0.007
		CS	8.496	LF	− 3.278*	= 0.007
	I	LF	7.772	CS	− 0.135	= 0.880
		CS	7.907	LF	0.135	= 0.880
51	O	LF	14.230	CS	0.027	= 0.976
		CS	14.203	LF	− 0.027	= 0.976
	D	LF	17.306	CS	4.715*	< 0.001
		CS	12.591	LF	− 4.715*	< 0.001
	I	LF	12.017	CS	− 1.738	= 0.053
		CS	13.755	LF	1.738	= 0.053
77	O	LF	13.901	CS	2.220*	= 0.014
		CS	11.681	LF	− 2.220*	= 0.014
	D	LF	16.725	CS	2.378*	= 0.049
		CS	14.347	LF	− 2.378*	= 0.049
	I	LF	11.987222	CS	− 0.815	= 0.363
		CS	12.801889	LF	0.815	= 0.363

### Correlation analysis between E and HIT

Pearson correlation coefficient was used to identify the correlation between E and HIT measured at different ages, in different layers and different parts. The results showed that both HIT and E were correlated with age (*P* < 0.05, *P* < 0.05) and location (*P* < 0.05, *P* < 0.05), while neither HIT nor E was correlated with layers (*P* > 0.05) (Table 8).

**Table 8:** The correlation coefficients between E and HIT by Pearson.

Parameter	Element	*P* value
HIT	Age	< 0.001
	Layer	= 0.586
	Location	< 0.001
E	Age	< 0.001
	Layer	= 0.525
	Location	= 0.011

## Discussion

Our results showed that HIT and E differed in all age groups, reaching the maximum in the middle-aged group and then slightly decreasing. The reason for this may be that human skeleton, including the skull, continuously grows and changes. From infants to young children, bone growth has a dominant role, after which it tends to enter a state of homeostasis for a period of time in adulthood, while bone quality and bone mass gradually decrease with age^22^. In this study, the correlation between age and layers was analyzed, and the result was positive, which further verified the correlation between each biomechanical parameter and age. Additionally, the difference in HIT for each layers was not statistically significant. E of diploe was higher than that of the inner plate and the outer plate. Further correlation analysis revealed no correlation between HIT/E and structure. This result might indicate that the osteon of the inner plate and the outer plate had the same or similar hardness as the trabecular meshwork constituting the diploe, and there was no difference in compression resistance.

Previous studies have reported differences in biomechanical properties of different parts of skulls. The main research method used in these studies was the three-point bending experiment of skull slices, and the research content was generally the detection of elastic modulus and bending strength. The results showed biomechanical differences among various parts, including cranial sutures^5^. In this study, the HIT and E of the left frontal and coronary sutures were studied using nanoindentation technology. Our results revealed a difference between the cranial and cranial sutures, and the HIT and E of the left frontal were higher than those of the coronary suture. Cranial suture continues to develop after birth, while it is not completely closed in infancy. With aging, the cranial tissues on both sides of the suture grow, continuously develop, and then gradually close^23^. In this study, the sampling location of the cranial suture was set as the skull adjacent to the cranial suture, which was different from the biomechanical parameters of the skull, thus indicatting that the biomechanical properties of the cranial tissue grown in the late period around the cranial suture were lower compared to other cranial tissues. Moazen et al. measured the biomechanical parameters of the mouse skull using nanoindentation experiment. Consistent with the results of this study, the elastic modulus of the mouse cranial suture was also lower than that of the skull^24^. These results indicated that the assignment of values to the cranial tissues around the cranial sutures should be made differently when the finite element model of the skull was constructed. Our study showed the correlation between HIT and E of the left frontal and coronary sutures with the change of age, indicating that each part of the skull grew to different degrees with aging, which was consistent with the results of the previous study^25,26^.

The results of this study showed that the thickness of LF/CS changed with ages. The peak was also in the middle-aged group and decreased to the elderly group, which was consistent with the change trend of HIT and E, coinciding with the research conclusion of Torimitsu et al., whose study showed that skull thickness correlated with biomechanical parameters^5^.

In summary, the present study successfully used the nanoindentation technique to measure the biomechanical parameters of various human skull layers from different parts of the human body at different ages. Our results revealed differences in the micromechanical parameters of the human skulls at different ages compared with the inner, diploe, and outer plates of the cranial sutures. The biomechanical properties of human skulls were related to age, location, and layers. Our research suggests that, when constructing the refined human head/skull finite element model, the properties and parameters of skull materials at different ages, in different parts (skull/cranial suture), and in different layers (outer plate, inner plate, and diploe) should be assigned with different values.

## Conclusion

The biomechanical properties of human skull/cranial suture with different ages and different layers are different. The HIT and E of skull are higher than those of cranial suture, and the diploe E is higher than the inner and outer plates. The differences of material mechanical parameters of age, layers and location should be considered in the finite element modeling of skull.
